# Unraveling the Relationship Between Vitamin D and Oxidative Stress: A Cross-Sectional Study

**DOI:** 10.7759/cureus.67818

**Published:** 2024-08-26

**Authors:** Jayballabh Kumar, Ashwani Sharma, Arkajit Dasgupta, Amrit Podder, Govindanagouda Naregal, Sariya Nazim, Hifzu Rehman

**Affiliations:** 1 Physiology, Teerthanker Mahaveer Medical College & Research Centre, Moradabad, IND; 2 Biochemistry, Teerthanker Mahaveer Medical College & Research Centre, Moradabad, IND; 3 Biochemistry, Shri B M Patil Medical College Hospital and Research Centre, BLDE (Deemed to be) University, Vijayapura, IND

**Keywords:** blood pressure, malondialdehyde, oxidative stress, reactive oxygen species, vitamin d

## Abstract

Introduction: Vitamin D, beyond bone metabolism, has played a significant role in various physiological processes, including modulation of oxidative stress and maintenance of vascular architecture. Oxidative stress, a state of altered balance between reactive oxygen species (ROS) and antioxidants, is a critical factor in the pathogenesis of various chronic diseases. Our study aims to explore the intricate relationship between serum vitamin D levels and markers of oxidative stress in normotensive and hypertensive individuals.

Materials and methods: A total of 108 age-matched participants (35 to 50 years) of both genders (54 males and 54 females) were included in this cross-sectional study according to the study design and assessed for their serum vitamin D level by using enzyme-linked immunosorbent assay (ELISA) method and serum malondialdehyde (MDA) level by using a spectrophotometer at 540 nm after measurement of the blood pressure. The data were entered in a Microsoft Excel sheet and analyzed using Statistical Package for Social Science (SPSS) software version 20.

Results: Our findings demonstrate a significant inverse correlation between serum vitamin D levels and MDA (r = -0.71, p < 0.001), indicating lower lipid peroxidation with higher vitamin D levels. Our study concludes by evident higher serum vitamin D levels associated with reduced oxidative stress, reflected by lower MDA.

Conclusion: These findings suggest a potential protective role of vitamin D against oxidative damage, which could have implications for the prevention of oxidative stress-related diseases.

## Introduction

Non-communicable diseases take the major bunk of the healthcare costs every year throughout the globe, and it is evident from the global numbers received in the last few years that more than one-third of total yearly deaths are directly or indirectly a resultant factor for cardiovascular diseases (CVDs) and the numbers of our country are nowhere behind it [1]. Oxidative stress, a condition that is developed due to the formation of reactive oxygen species (ROS) in excessive amounts along with inadequate antioxidant defenses, is contributing to cellular damage and the pathogenesis of various chronic diseases, including CVD, diabetes, and neurodegenerative disorders [2]. There were years when the scientific community thought that everything was sorted out with vitamin D with the discovery of its role in rickets but the relationships that were established in the previous years for vitamin D with other diseases, especially the diseases of the cardiovascular system, it is quite obvious now that the pioneering studies just demonstrated the tip of the iceberg and there are a lot to be explored with vitamin D and its involvement in various morbidities and mortalities [3]. Vitamin D, as the only hormone in its active state (1,25-dihydroxycholecalciferol) responsible for the absorption of calcium from the gastrointestinal tract, traditionally known for its role in calcium homeostasis and bone health, has been increasingly recognized for its potential antioxidative properties in the previous years [4]. It has also drawn attention among researchers in medicine and physiology in such a way that newer dissections are being made every month worldwide for its involvement in newer discoveries [5]. Our study investigates the relationship between the levels of serum total vitamin D and parameters of oxidative stress in the middle-aged population.

## Materials and methods

Study design

Our study is a prospective cross-sectional study.

Study setting

The study was conducted in Vijayapura district of Karnataka, India, after we received the clearance from the Institutional Ethical Committee of BLDE (Deemed to be University).

Sample size

A total of 108 age-matched (35 to 50 years) participants of both genders (54 males and 54 females) were included in our study and divided into three groups following voluntary informed written consent was obtained.

Method of collection of data

According to the latest American Heart Association (AHA) guidelines, we recruited normotensive individuals in group 1, individuals with stage 1 hypertension in group 2, and individuals with stage 2 hypertension in group 3 [6]. All the included participants of each group belonged to the age group of 30 to 55 years, and each group consisted of 36 participants which included 18 males and 18 females. We have excluded the participants of any chronic diseases, smokers, alcoholics, drug abuse, taking any medications that alter the vasculature, current supplementation of vitamin D or antioxidants, and recent acute illness. The blood pressure was measured by using a manually calibrated digital sphygmomanometer between 8 AM to 9 AM by following the AHA guidelines [7]. After the blood pressure was measured, all the participants were given rest for 15 minutes following which 5 ml venous blood samples were collected in a plain vial and centrifuged at 3,500 rpm for 10 minutes. The serum samples were collected and stored at -21 degrees Celsius for a maximum of 21 days and analyzed for serum vitamin D level and serum malondialdehyde (MDA) level. The vitamin D level was analyzed by enzyme-linked immunosorbent assay (ELISA) method, and the serum MDA was analyzed by using a spectrophotometer at 540 nm.

Sampling strategy

We have opted for the comprehensive sampling strategy for our study where all the participants underwent a comprehensive clinical evaluation, including demographic data, medical history, and lifestyle factors.

Statistical analysis

The obtained data were entered in a Microsoft Excel sheet and were analyzed using SPSS software version 20. The data was presented as mean ± standard deviation (SD) after performing the t-test. Spearman’s correlation was performed to obtain the correlation coefficients (r-value) for the relationships between serum vitamin D levels and serum MDA levels. All the performed tests were two-tailed, and p <0.05 was considered statistically significant.

## Results

Table 1 depicts the data of comparison of different study parameters that were investigated during the study period. As all the components of blood pressure are significantly higher in group 2 than in group 1 and in group 3 than in group 2, respectively, it is evident that the proper study design was followed for the segregation of participants for the study. It is quite obvious from the results that there is a significant (p<0.05) increase in the serum MDA level in group 2 participants than in group 1 participants and similar results are also visible in group 3 when compared with group 2. Conversely, the serum vitamin D levels were found to be increased in group 2 participants than in group 3 participants and in group 1 participants than in group 2 participants.

**Table 1:** Comparison of all investigated parameters between the groups Data is represented in the form of mean ± SD. p ≤ 0.05 is taken as statistically significant. SBP, systolic blood pressure; DBP, diastolic blood pressure; PP, pulse pressure; MAP, mean arterial pressure; MDA, malondialdehyde; Group 1, normotensive individuals; Group 2, stage 1 hypertensive individuals; Group 3, stage 2 hypertensive individuals. The p values are between all the three groups. The t-test was performed to get the values.

	Group 1 (n=36)	Group 2 (n = 36)	Group 3 (n = 36)	p-value
SBP (mmHg)	114.94 ± 3.50	134.17 ± 2.76	148.72 ± 9.17	<0.0001
DBP (mmHg)	72.61 ± 5.34	83.17 ± 3.62	90.67 ± 6.27	<0.0001
PP (mmHg)	42.33 ± 5.52	51.00 ± 4.62	58.06 ± 10.89	<0.0001
MAP (mmHg)	86.72 ± 4.04	100.17 ± 2.56	110.09 ± 5.28	<0.0001
Vitamin D (ng/ml)	35.92 ± 4.15	25.44 ± 3.97	17.09 ± 3.45	<0.0001
MDA (μmol/L)	0.99 ± 0.19	1.39 ± 0.23	2.08 ± 0.45	<0.0001

**Figure 1:**
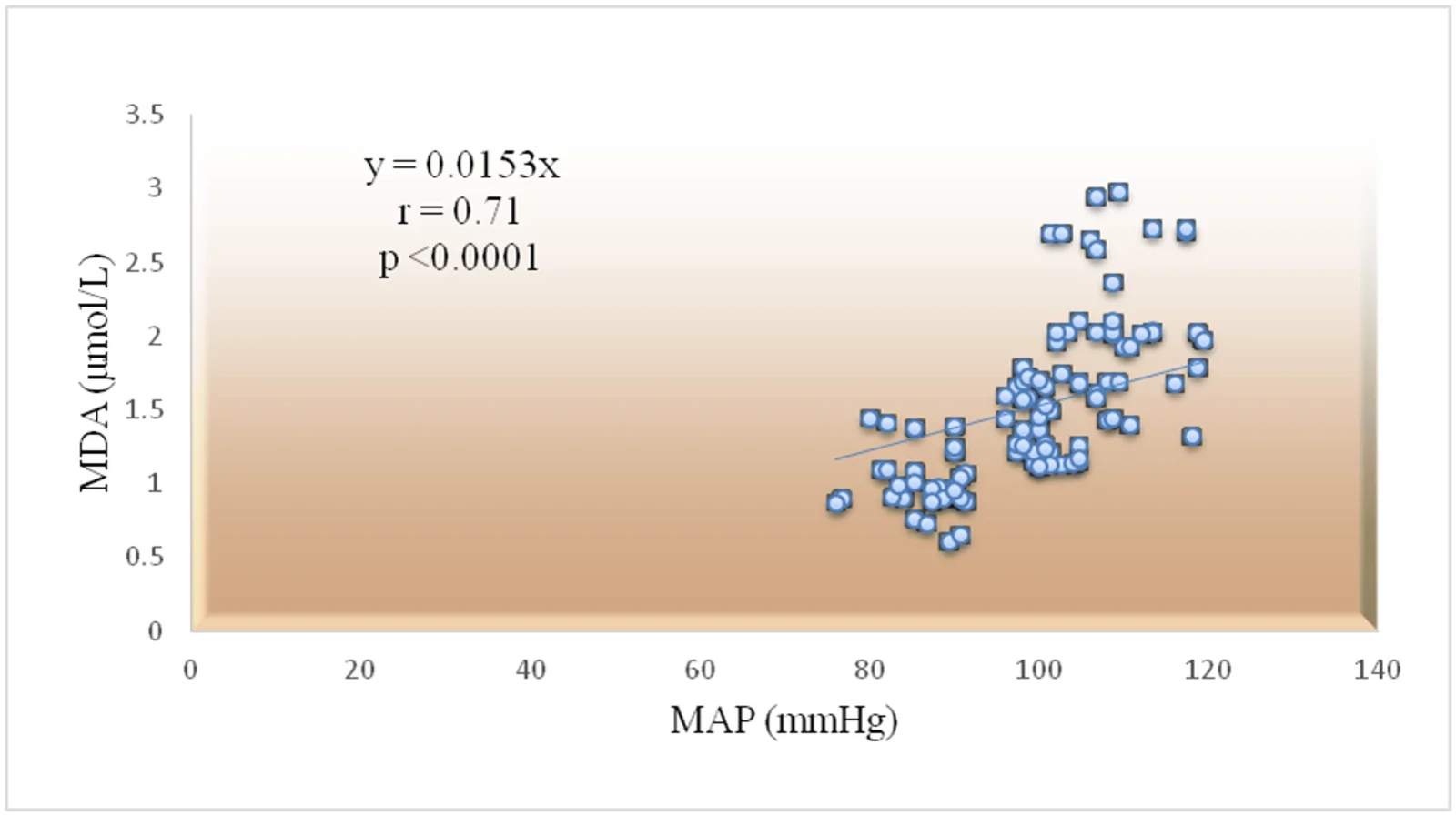
Correlation of MAP with MDA r, correlation coefficient; p ≤ 0.05 is considered as statistically significant; MDA, malondialdehyde; MAP, mean arterial pressure. The Spearman's correlation was performed to get the values.

Table 2 shows the correlation of all the investigated parameters with vitamin D in the total study population. We found a significant (p<0.05) negative correlation of all the study parameters with vitamin D. It is quite obvious from the results that oxidative stress is going to be moderately decreased with an increased amount of vitamin D in the blood. Although we found a negative correlation of vitamin D with all the components of blood pressure, the highest correlation coefficient value was found for mean arterial pressure (MAP), making MAP the most trustworthy parameter to be considered among all the components of blood pressure. While analyzing, we also found a positive correlation between serum MDA levels with MAP as evident from Figure 1. All the data were age matched, and no significant (p>0.05) difference was found when the data were compared between the genders in each group or in the entire study population.

**Table 2:** Correlation of all the investigated parameters with vitamin D r, correlation coefficient; p ≤ 0.05 is considered as statistically significant; SBP, systolic blood pressure; DBP, diastolic blood pressure; PP, pulse pressure; MAP, mean arterial pressure; MDA, malondialdehyde. The Spearman's correlation was performed to get the values.

	Vitamin D (ng/ml)	
	Total study population (n=108)	p-value
SBP (mmHg)	r = -0.83*	<0.0001
DBP (mmHg)	r = -0.78*	<0.0001
PP (mmHg)	r = -0.56*	<0.0001
MAP (mmHg)	r = -0.85*	<0.0001
MDA (μmol/L)	r = -0.71*	<0.0001

## Discussion

Our results depict that oxidative stress has a significant role in increasing blood pressure, and decreasing the amount of vitamin D may be associated with it indicating that higher vitamin D levels are associated with lower lipid peroxidation which might result in increased arterial stiffness [8,9]. The results from our study also suggest an enhanced antioxidant defense in individuals with higher vitamin D levels as MDA levels are decreased with increased levels of vitamin D. This study provides evidence of a significant relationship between serum vitamin D levels and oxidative stress markers. Higher vitamin D levels are associated with reduced lipid peroxidation and enhanced antioxidant enzyme activities [10]. These findings support the hypothesis that vitamin D may exert protective effects against oxidative stress, potentially contributing to the prevention of oxidative stress-related diseases. The following mechanisms may also play a significant role in the decrease in vitamin D levels with an increase in oxidative stress with an increase in blood pressure such as endothelial dysfunction, inflammation, arterial stiffness, and advanced vascular age.

Endothelial dysfunction and inflammation

A proper endothelial function is necessary for maintaining a proper vascular architecture. Under pathogenic conditions, the excessive production of ROS not only facilitates the degradation of nitric oxide (NO), a potent vasodilator but also suppresses the synthesis of it. As a result, there is less vasodilation which in turn facilitates the oxidative stress by increasing the free radicals. The presence of vasoprotective agents like vitamin D might help delay the effects to an extent by acting as an antioxidant, whereas insufficient levels of it will aggravate the ongoing pathogenesis of the vasculature. Pro-inflammatory factors like tumor necrosis factor α (TNF-α) and interleukin 6 (IL-6) are precipitating factors for the development of endothelial dysfunction in addition to ROS which restrain the bioactivity of nitric oxide and nitric oxide synthase by upregulating various atherosclerotic factors expression through the nuclear factor kappa-light-chain-enhancer of activated B cells (NF-κB) pathway. Vitamin D inhibits these pro-inflammatory activities by suppressing NF-κB signaling [11].

Vascular stiffness

Stiffening of the vessels alters the architecture inside it which in return aggravates the molecular biology of the endothelium in such a way that the result is the development of diminished or decreased elasticity of the vessels [12,13]. It precipitates the pathway for ROS formation and thereby increases oxidative stress and hypertension [14]. Vitamin D insufficiency plays a significant role in this process as decreased antioxidant properties inside vessels guard the pro-inflammatory mechanisms to stimulate. Vitamin D influences the optimal functioning of the endothelium and smooth muscle cells by modulating the renin-angiotensin-aldosterone axis (RAAS). Insufficient vitamin D levels in the blood may result in increased pulse wave velocity (PWV), a highly sensitive marker to measure arterial stiffness, which as a result causes the remodeling of the cardiovascular system and helps in the development of CVD, and increased vascular stress due to the lack of antioxidants may precipitate the ongoing pathogenic mechanisms [15].

Advanced vascular age

The presence of vitamin D receptors in almost all human cells and tissues indicates its role in the advancement of vascular age due to its deficiency or insufficiency by modulating the immunological system and the cardiovascular system. Advancement in vascular age is a resultant factor from the increase in free radical activity in the body altering the homeostatic mechanisms. As 90% of the production of vitamin D is dependent on its production from cutaneous origin, it is quite obvious that the antioxidant property has to be compensated by supplementation in either synthetic form or with dietary sources, if a person is not properly exposed to the sunlight due to lifestyle obligations [16]. Several lifestyle modifications can help in reducing oxidative stress such as proper optimal sunlight exposure, performing yoga and breathing exercises, supplementation of vitamin D, and induction of anti-inflammatory agents [16].

Role of sunlight exposure in reduction of oxidative stress

The main pathway for generating the active form of vitamin D in humans occurs in the epidermal layer of the skin via an intricate physicochemical process when ultraviolet (UV) rays are absorbed, and thereby, almost 90% of all the requirements of the vitamin D in humans originate from this cutaneous production. The maximum concentrations of vitamin D3 occur 24-48 hours after UV-B exposure. Once it reaches the bloodstream, the antioxidant mechanisms of it initiate the scavenging mechanisms by removing the free radicals which in return helps in decreasing the oxidative stress. Thus, it is advisable to prefer the early morning sunlight exposure rather than the afternoon period as UV-B radiation remains at its peak during the early morning hours [17,18].

Role of yoga in reduction of oxidative stress

Different types of practices in yoga such as breathing exercises, posture-maintaining techniques, etc. play a major role in decreasing oxidative stress as they initiate the mechanisms to increase the levels of serum endothelial nitric oxide synthase (eNOS) which in turn increases the level of serum nitric oxide (NO). As it is a well-established fact that the higher potency of serum nitric oxide as a vasodilator modulates the vasculature to decrease the stiffening of the vessels, in return this mechanistic pathway also helps in reducing the oxidative stress by inhibition of ROS and thus reduces the prevalence of the morbidities originates from the CVD [18,19].

Supplementation of vitamin D

Vitamin D supplementation should be initiated in patients if the vitamin D concentration is <20 ng/ml. Guidelines from the Endocrine Society Clinical Practice suggest supplementation of vitamin D in different age groups which may help reduce the burden of its deficiency and insufficiency [20, 21].

Limitations of the study

Our study is conducted with a very small sample size which we consider as one of the limitations of our study. The data after providing vitamin D supplementation might show a different scenario which we consider the other limitation of our study.

## Conclusions

Our study dissects a novel link between serum levels of total vitamin D status and parameters of oxidative stress, suggesting that maintaining adequate vitamin D levels could be a strategic approach to mitigate oxidative damage. Vitamin D supplementation along with lifestyle modifications can play a vital role in the reduction of oxidative stress and in maintaining proper cardiovascular health. We conclude our study by establishing the beneficial role of vitamin D in the reduction of oxidative stress and the improvement of cardiovascular health. Further interventional studies are expected to establish the causality and elucidate the underlying mechanisms.
