# Correction: Unraveling the Relationship Between Vitamin D and Oxidative Stress: A Cross-Sectional Study

**DOI:** 10.7759/cureus.c487

**Published:** 2026-10-06

**Authors:** Jayballabh Kumar, Ashwani Sharma, Arkajit Dasgupta, Amrit Podder, Govindanagouda Naregal, Sariya Nazim, Hifzu Rehman

**Affiliations:** 1 Physiology, Teerthanker Mahaveer Medical College & Research Centre, Moradabad, IND; 2 Biochemistry, Teerthanker Mahaveer Medical College & Research Centre, Moradabad, IND; 3 Biochemistry, Shri B M Patil Medical College Hospital and Research Centre, BLDE (Deemed to be) University, Vijayapura, IND

Dr. Mohammad Kamran Iqbal has been removed from the author list at his request, as he stated that he had not consented to authorship. The submitting author maintains that verbal consent was given; all remaining authors agreed to the removal.

